# Retraction: Multi-Level Interactions between the Nuclear Receptor TRα1 and the WNT Effectors β-Catenin/Tcf4 in the Intestinal Epithelium

**DOI:** 10.1371/journal.pone.0228994

**Published:** 2020-02-05

**Authors:** The PLOS ONE Editors

Following the publication and correction of this article [1, 2], additional concerns have been raised regarding Fig 2 and Fig 5:

- In Fig 2C, lane 2 and 3 in the activated β-catenin panel, representing the T3 and the T3-Wnt3a samples, appear similar.
- In Fig 2C, lane 1 and 3 in the Actin panel, representing the Ctrl and the T3-Wnt3a samples, appear similar.
- In Fig 2C, vertical discontinuities in background have been detected between lane 3 and lane 4 of the Activated β-catenin panel, and between lane 1 and lane 2 in the Actin panel.
- In Fig 5, the T3- and T3+ results for the WB:TRα1 Inputs panel appear similar.
- In Fig 5, lanes 3, 4 (TRα1 IP) appear similar to lanes 5, 6 (β-catenin IP) in the WB:β-catenin panel.
- In Fig 5, the T3- and T3+ results for the WB:β-catenin Inputs panel appear similar.
- In Fig 5, irregularities have been detected in background granulation just below the Inputs (+) band of the WB: β-catenin panel.

The authors have indicated that the underlying primary data for the figures above are no longer available.

In light of the concerns affecting multiple figure panels that question the integrity of these data, the *PLOS ONE* Editors retract this article.

MS, SS, INL, JN, DA did not respond. MP did not agree with retraction.
